# How the presence of residual lipids in a yellow mealworm protein concentrate affects its foaming properties?

**DOI:** 10.1016/j.crfs.2024.100763

**Published:** 2024-05-07

**Authors:** Ugo Berthelot, Juliette Barrot, Gwenn Pinel, Alain Doyen

**Affiliations:** Department of Food Sciences, Institute of Nutrition and Functional Foods (INAF), Université Laval, Québec (QC), Canada, G1V 0A6

**Keywords:** *Tenebrio molitor*, Protein concentrate, Lipid content, Residual lipids, Foaming properties

## Abstract

The use of whole and visible insects is poorly accepted in Western countries, and this remains a significant challenge for product development. However, using insect-based protein-rich ingredients, like protein concentrate, can improve levels of consumer approval. The residual lipid content in insect protein concentrates can influence their techno-functional properties. Our study therefore aimed to evaluate the impact of the residual lipid content on the protein structure and foaming properties of a mealworm protein concentrate. Our results showed that the protein content increased from 78.01 to 84.82 % after using chloroform-methanol for lipid removal. The particle size distribution shifted from a bimodal to a unimodal pattern, and the surface hydrophobicity decreased from 267.02 to 48.91 after completely removing lipids by chloroform-methanol, with no noticeable impact on the protein profile. The foaming capacity improved, resulting in the formation of a firm and fluffy foam with high stability over time. These results highlight the importance of controlling the residual lipid content in mealworm protein concentrates to enhance their techno-functional properties. The next steps will entail comprehensively characterizing the lipid profile and exploring the various mechanisms contributing to the techno-functional properties.

## Highlights

• Residual lipids modified the particle-size distribution of mealworm protein extract.
• Residual lipids removal reduced the protein surface hydrophobicity.
• Foaming properties were largely improved after residual lipids removal.

## Graphical abstract

**Figure:**
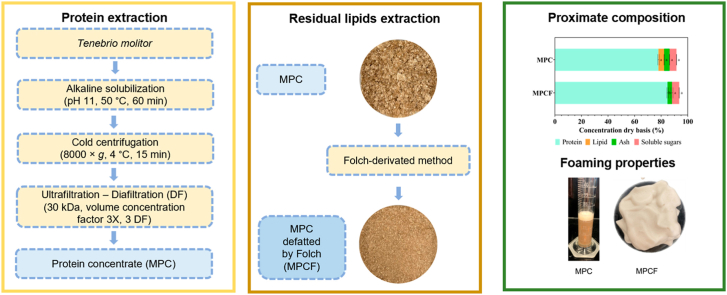

## 1 Introduction

Edible insects provide a potentially sustainable alternative protein source due to their minimal environmental impact and nutritional values (Akhtar and Isman, 2018; van Huis, 2016). Utilizing insect-based protein ingredients in the development of innovative food formulations, instead of incorporating whole and visible insects, is preferred to enhance consumer approval (La Barbera et al., 2018). Producing insect protein concentrate requires a defatting step followed by a protein extraction step (Indriani et al., 2021). Various solvents (Gravel et al., 2021; Laroche et al., 2019; Ravi et al., 2019) and physical techniques (Boukil et al., 2022; Laroche et al., 2019; Purschke et al., 2017) are used to extract fat from insects. However, some residual lipids, predominantly polar lipids and phospholipids, remain after extraction and may still be present in the defatted insect meal and in the resulting protein concentrate (Choi et al., 2017). The presence of this residual lipid fraction can affect the techno-functional properties of the protein-rich ingredient, as previously demonstrated in dairy-based protein products (Abd El-Salam et al., 2009; Oliveira and O’Mahony, 2020) and chickpeas (Sánchez-Vioque et al., 1998). Using chloroform-methanol instead of hexane has improved the extraction of polar and non-polar lipids from the mealworm and black soldier fly meals (Tognocchi et al., 2023). Our study therefore aimed to compare the proximate composition, protein profile, structure, and foaming properties of two different mealworm protein concentrates: one with a residual lipid fraction and the other with no detectable residual lipids.

## 2 Materials and methods

### 2.1 Raw materials

Live mealworm larvae (*Tenebrio molitor*) were kindly provided by Groupe Neoxis Inc. (Saint-Flavien, Quebec, Canada). A blanching treatment at 75 °C for 20 min was performed. The blanched larvae were frozen at −30 °C, freeze-dried and ground into a powder (Chopper 5KFC3515, KitchenAid, USA). All other reagents and chemicals were purchased from MilliporeSigma (Burlington, MA, United States) and were of analytical grade.

### 2.2 Methods

#### 2.2.1 Protein extraction

Yellow mealworm protein concentrate (**MPC**) was produced by ultrafiltration-diafiltration (UF-DF) as described by Berthelot et al. (2024). Briefly, an alkaline extraction at pH 11 was performed for 1 h on the meal. This was followed by a cold centrifugation (8000×*g*, 15 min, 4 °C) to remove the fat (top layer) and the insoluble phase (pellet), leaving the supernatant, which was then freeze-dried. Subsequently, the protein extract was suspended in deionized water to reach a protein concentration of 2 % (w/v). The solution was then concentrated until a volume concentration factor (VCF) of 3X was reached, as determined by the weight (kg) of the permeate collected. Then, diafiltration was applied to the retentate, which was diluted with 2 diavolumes (DV) of deionized water (4 °C) before initiating another concentration cycle (VCF of 3X). Two more cycles were performed for a total of three DF. The final retentate was freeze-dried to obtain the mealworm protein concentrate.

#### 2.2.2 Lipid extraction

The residual lipid content in the MPC was extracted according to Tognocchi et al. (2023), with some modifications. Briefly, 12 g of the mealworm protein concentrate was mixed with 80 mL of chloroform-methanol (1:1) solvent and stirred manually for 30 s. The resulting suspension was incubated at 60 °C for 20 min, and then 40 mL of chloroform was added to obtain a ratio of 2:1 chloroform-methanol. After Buchner filtration with Whatman #4 paper (20–25 μm), the residue and the filtrate were collected. The extraction with chloroform-methanol was repeated two more times (for a total of three extractions) on the residue to maximize lipid extraction. Afterwards, the residue was recovered, left overnight inside a fume hood for solvent evaporation, and stored at −20 °C for further analyses. The residue was identified as the mealworm protein concentrate after complete lipid removal (**MPCF**). Filtrates recovered after each lipid extraction were combined, and a 0.58 % NaCl solution was added in a 1:5 ratio to the volume of the filtrates. This was done to generate a biphasic system for the removal of non-lipid substances (Folch et al., 1957). The solution was centrifuged (4500×*g*, 20 °C, 5 min) to enhance the separation of aqueous and organic phases. The aqueous phase was discarded. The organic phase was then recovered and evaporated using a rotary evaporator (R-215, Büchi, Flawil, Switzerland). After evaporation, the extract, which was mainly composed of lipids, was weighted, and discarded.

### 2.3 Analysis

#### 2.3.1 Proximate composition

The protein content of the MPC and the MPCF was measured using the Dumas combustion method (Elementar rapid Micro N cube, Langenselbod, Germany) with a nitrogen-to-protein conversion factor of 6.25 (Berthelot et al., 2024). The lipid content was determined using the Soxhlet method with hexane as described by Laroche et al. (2019). Moisture and ash contents were obtained by AOAC 950.46 (A) and 920.153, respectively. The soluble sugar content was determined as described by Pinel et al. (2024).

#### 2.3.2 Protein profile by SDS-PAGE

The protein profiles of both the MPC and the MPCF were obtained by sodium dodecyl sulphate-polyacrylamide gel electrophoresis (SDS-PAGE) under non-reducing and reducing conditions as described by Boukil et al. (2022) with slight modifications. Solutions containing 1 % of proteins (w/v) for each concentrate were prepared. For the non-reducing condition, a volume of 25 μL of Laemmli 2 × buffer (Bio-Rad, Mississauga, ON, Canada) was added to 25 μL of each sample, and 10 μL was then loaded into each well. For the reducing condition, a volume of 25 μL of buffer (5 % 2-mercaptoéthanol with 95 % Laemmli 2 × ) was added to 25 μL of each sample. The solutions were then immersed in boiling water for 5 min and then cooled on ice for 10 min before loading 10 μL into each well. For both conditions, electrophoresis was carried out using a 12 % TGX Stain-Free polyacrylamide gel (Bio-Rad, Mississauga, ON, Canada), cast and run in a 1 × tris-glycine SDS buffer (Bio-Rad, Mississauga, ON, Canada) at 15 mA at room temperature until the migration was complete. The proteins were subsequently stained with Coomassie blue (1 g/L of Coomassie Brilliant Blue R-250, 10 % acetic acid, 40 % ethanol and 50 % water) for 1 h and then destained with a solution of 10 % methanol and 10 % acetic acid for 1 h. The molecular weight (MW) was estimated using MW markers (Precision Plus Protein™ 161–0363 All Blue Unstained Protein Standards, Bio-Rad, Mississauga, ON, Canada). Gel images were taken using the ChemiDoc™ MP Imaging System (ChemiDoc MP, Bio-Rad, Mississauga, ON, Canada).

#### 2.3.3 Surface hydrophobicity

The surface hydrophobicity was measured according to the protocol of Pinel et al. (2024). Briefly, the MPC and the MPCF solutions, with protein concentrations ranging from 0.01 % to 0.05 %, were prepared in 2 mM phosphate buffer at pH 7. Relative fluorescence units (RFU) of the samples with the addition of 8-anilino-1-naphthalene sulfonate (ANS) (8 mM ANS in 0.1 M phosphate buffer at pH 7) (RFU_protein + ANS_) or without (RFU_protein_) were measured using the modular multimode microplate reader BioTek Synergy H1 (Agilent Technologies, Santa Clara, CA, USA). The fluorescence intensity of each protein solution series was measured using an excitation wavelength of 380 nm and an emission wavelength of 460 nm. The surface hydrophobicity index (*H*_*0*_) was calculated using the slope of the linear regression analysis of fluorescence intensity as a function of the protein concentration.

#### 2.3.4 Foaming properties

The foaming capacity (FC) and foam stability (FS) were determined as described by Pinel et al. (2024) with slight modifications. Briefly, the MPC and the MPCF protein solutions (3 % w/v) were prepared in deionized water at pH 4 and 9. The suspensions were then whipped using a hand mixer (KitchenAid, KHM512IB, Benton Charter Township, MI, USA) at the maximum speed (1130 rpm) for 4 min and then immediately transferred into a cylinder. The volume of foam was recorded at time 0 (*V*_*0*_), and then at 15-min intervals for a total of 45 min (*V*_*t*_). The volume of liquid (*V*_*liq*_) was also noted. The FC and FS values were calculated using equations (1), (2):

(1) $$FC\;\left(\%\right)=\frac{V_{0\;}-\;V_{liq}}{V_{liq}}\times 100$$

(2) $$FS\;\left(\%\right)=\frac{V_{t}\;-\;V_{liq}}{V_{0}\;-\;V_{liq}}\times 100$$

#### 2.3.5 Particle size analysis

The particle-size distribution of the concentrates was measured by laser light scattering using a Mastersizer 3000 analyser (Malvern Mastersizer 3000, Malvern Instruments, UK). MPC and MPCF suspensions (5 % protein *w/v*) were prepared, and the pH was adjusted to 9 using 0.1 M NaOH. Suspensions were then centrifuged at 18,000×*g* for 15 min at 20 °C to remove insoluble aggregates. The resulting supernatants were added to the Mastersizer 3000 Hydro SV dispersion unit until an obscuration of 5 % was reached. Particle and dispersant refractive indexes were set at 1.48 and 1.33, respectively, according to Boukil et al. (2022). The particle size distribution was expressed in volume density (%).

### 2.4 Statistical analysis

Statistical analysis was performed using R® 4.2.1 software (R Core Team, 2022). All experiments were conducted in duplicate. The results were expressed as the mean ± standard deviation. The proximate composition and surface hydrophobicity data were subjected to a one-way analysis of variance (ANOVA) while foaming properties were subjected to a two-way ANOVA. The differences in the means between the samples were assessed using the Tukey test, and p-values of <0.05 were considered statistically significant.

## 3 Results

### 3.1 Proximate composition and surface hydrophobicity of the mealworm protein concentrates

Table 1 presents the proximate composition and surface hydrophobicity (*H*_*0*_) of the MPC and the MPCF. After lipid extraction by chloroform-methanol, the protein content increased in the MPCF compared with the MPC, with respective values of 84.82 and 78.01 %. No significant differences were observed in the soluble sugars or in ash content after lipid extraction. The lipid content in MPC was higher after lipid extraction using chloroform-methanol (13.66 %) compared with the Soxhlet method with hexane (4.31 %). After the complete lipid removal, no lipids were detected in the MPCF. After removing the residual lipids, the surface hydrophobicity index decreased substantially. Specifically, the H_0_ value of the MPCF decreased to 48.91 a.u., while the H_0_ value of the MPC was 267.02 a.u.

**Table 1:** Composition expressed on a dry matter basis and the surface hydrophobicity index (H_0_) of the MPC and the MPCF.

	Protein	Soluble sugars	Lipid (hexane)	Lipid (chloroform-methanol)	Ash	H_0_
	% (w/w)					
MPC	78.01 ± 0.50^a^	5.24 ± 0.08^a^	4.31 ± 0.29^a^	13.66 ± 1.90^a^	3.98 ± 0.10^a^	267.02 ± 24.55^a^
MPCF	84.82 ± 1.27^b^	5.44 ± 0.17^a^	nd	ND	3.31 ± 0.78^a^	48.91 ± 6.55^b^

Different superscript letters within a column denote statistically significant differences between samples (p < 0.05). nd: not determined due to low amount of samples. ND: not detected.

### 3.2 Protein profile, particle size and foaming capacities

**Fig. 1:**
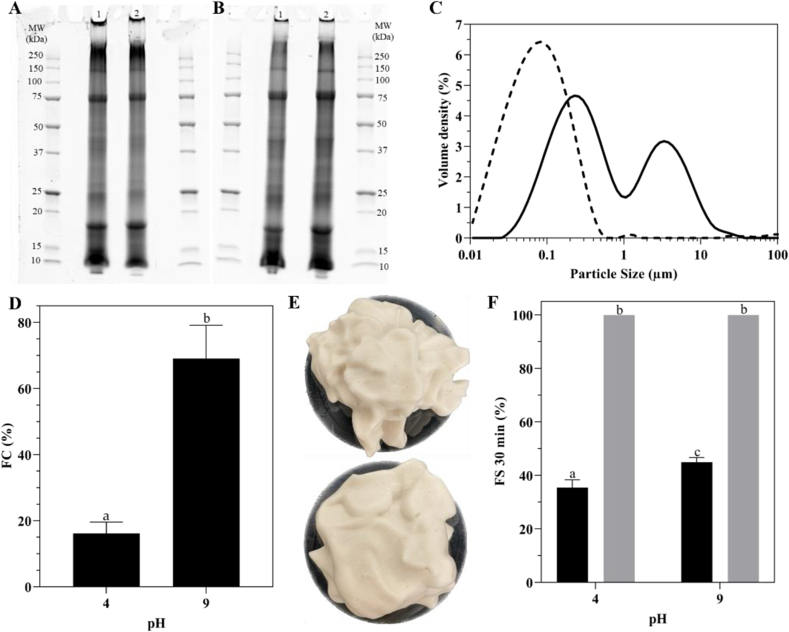
MPC (lane #1) and MPCF (lane #2) protein profiles generated by SDS-PAGE under non-reducing (A) and reducing (B) conditions. The particle size distribution of the MPC (solid line) and the MPCF (dashed line) (C). The foaming capacity of the MPC at pH 4 and 9 (D). Photos (E) of the MPCF foam obtained at pH 4 (Top) and pH 9 (Bottom). MPC (dark) and MPCF (grey) foaming stability at 30 min at pH 4 and 9 (F).

Fig. 1 presents the protein profiles analyzed by SDS-PAGE, particle size distribution, foaming capacities, and the photos of the foams obtained for both the MPC and the MPCF. Similar electrophoretic protein patterns were obtained for both concentrates (Fig. 1A and B). Specifically, under non-reducing conditions (Fig. 1A), high molecular weight (MW) proteins (>250 kDa) and large and intense low MW bands (<10 kDa) were detected for both the MPC and the MPCF. Additionally, prominent bands with MWs close to 150, 75, 18, and 10 kDa were observed in both the MPC and the MPCF. Under reducing conditions, the intensity of the bands corresponding to high MW proteins (>250 kDa) decreased, while it increased for other bands. As presented in Fig. 1C, two different particle populations (∼0.23 μm and 2.93 μm) were obtained in the MPC, whereas a single major population (∼0.08 μm) was obtained in the MPCF after chloroform-methanol lipid extraction. The foaming capacity (FC) of the MPC was low at pH 4 (16.2 %), but it was significantly increased at pH 9 (69.1 %). The removal of residual lipids resulted in greatly improved the foaming properties, resulting in a firm and fluffy foam at both pH levels (Fig. 1E). However, the foam was too dense to calculate a FC value. The foaming stability (FS) of the MPCF at both pH levels after 30 min was higher than that of the MPC, reaching 100 % stability (Fig. 1F). The foaming stability of MPC was greater at pH 9, with a value of 45%, than at pH 4, where it was 35.4 % (Fig. 1F).

## 4 Discussion

As expected, the removing the residual lipids from the mealworm protein concentrate using chloroform-methanol increased protein levels, consistent with previous reports (Jeong et al., 2021). Importantly, the electrophoretic protein profiles remained similar, confirming that chloroform-methanol had no impact on protein content or aggregation. The significantly higher lipid content obtained with chloroform-methanol indicates that hexane did not facilitate a complete lipid extraction from the mealworms. This result is consistent with the findings of Dos Santos et al. (2015), which demonstrated a higher lipid extraction rate in *Chlorella vulgaris* biomass using chloroform-methanol (2:1) compared to hexane. A decrease in the surface hydrophobicity of the mealworm protein concentrate was observed after the residual lipids were removed. This result aligns with the findings by Kim et al. (2021) on protein extract from *Protaetia brevitarsis* larvae. The observed decrease in surface hydrophobicity could be explained by protein structure modifications induced by the use of organic solvents (Martínez-Aragón et al., 2009), as previously reported in soy protein extracts (L'Hocine et al., 2006). However, Gravel et al. (2023) reported no differences in the surface hydrophobicity of a pea protein isolate generated from defatted pea flour using hexane. Differences in the protein origin (e.g., globular proteins from peas and muscular proteins from mealworms) and extraction solvents (e.g., hexane and chloroform-methanol) could account for the variation in findings. In addition, it is well-known that mealworm is composed of lipoproteins, mainly lipophorins (Jones et al., 1972), and that chloroform-methanol was demonstrated as efficient to remove lipoproteins from different matrices (Reis et al., 2013; Saini et al., 2021). Consequently, it could be also possible that a part of these or all lipoproteins were removed from the MPC and recovered in the lipid fraction. The particle-size distribution in the mealworm protein concentrate with residual lipids differed slightly from that reported by Boukil et al. (2022). The proportion of the second population peak in their study was lower than in this study. This difference can be explained by the disparity in the residual lipid content (0.51% in Boukil et al. (2022) and 4.31 % in this study), as it is well-known that the presence of lipids can influence particle-size distribution (Bolenz and Manske, 2013; Olson et al., 2004). Following lipid removal, substantial changes were observed in the particle size dispersion, transitioning from a bimodal to a unimodal distribution with the absence of the second peak. This result could indicate that the second peak corresponded to lipids or protein bound with lipids which disappear after lipid removal, while the shift of the first peak was likely due to the rearrangement of proteins with a denser structure. This finding is consistent with the results of Gravel et al. (2023), which described a decrease in the particle-size distribution following a defatting step of a pea protein isolate by hexane. Removing the residual lipid fraction by chloroform-methanol substantially enhanced the foaming properties of the resulting mealworm protein concentrate, resulting in the formation of a firm and fluffy foam. Previous reports suggest that lipid content significantly affects the foaming properties of whey protein concentrates (Karleskind et al., 1995; Vaghela and Kilara, 1996), pulses (Gravel et al., 2023; Toews and Wang, 2013), and insects (Borremans et al., 2020; Gravel et al., 2021). Moreover, a study by Zayas (1997) indicated that even small lipid contents (e.g., less than 0.1 %) can impact protein foaming properties. Residual lipids could affect protein foaming properties by interfering with the formation and stability of foam and by preventing proteins from organizing themselves around air bubbles (Zayas, 1997). Furthermore, residual lipids may decrease surface elasticity, thereby increasing the likelihood of air bubbles coalescence which in turn reduces the foam stability (Denkov, 2004; Denkov and Marinova, 2006).

## 5 Conclusion

The complete removal of residual lipids from a mealworm protein concentrate led to a modified particle size distributions and decreased surface hydrophobicity. It also significantly improved protein foaming properties, leading to a firm and fluffy foam with high stability. It is therefore necessary to consider the residual lipid content when assessing the foaming properties of a mealworm protein concentrate. The effects of removing lipids from mealworm protein concentrate could also extend to other techno-functional properties, such as emulsification.

## Funding

This research was funded by the Innov'Action research program of the 10.13039/100008777MAPAQ (Ministry of Agriculture, Fisheries and Food, Government of Quebec) (IA222729).

## CRediT authorship contribution statement

**Ugo Berthelot:** Conceptualization, Formal analysis, Data curation, Writing – original draft, Writing – review & editing. **Juliette Barrot:** Formal analysis, Data curation. **Gwenn Pinel:** Formal analysis, Data curation, Writing – review & editing. **Alain Doyen:** Conceptualization, Resources, Supervision, Writing – review & editing.

## Declaration of competing interest

The authors declare that they have no known competing financial interests or personal relationships that could have appeared to influence the work reported in this paper.

## Data Availability

Data will be made available on request.
